# The effects of group and single housing and automated animal monitoring on urinary corticosterone levels in male C57BL/6 mice

**DOI:** 10.14814/phy2.12703

**Published:** 2016-02-11

**Authors:** Remi Kamakura, Miia Kovalainen, Juhani Leppäluoto, Karl‐Heinz Herzig, Kari A. Mäkelä

**Affiliations:** ^1^ Research Unit of BiomedicineFaculty of MedicineUniversity of OuluOuluFinland; ^2^ Biocenter of Oulu and Medical Research Center Oulu and Oulu University HospitalUniversity of OuluOuluFinland; ^3^ Department of Gastroenterology and MetabolismPoznan University of Medical SciencesPoznanPoland

**Keywords:** stress, metabolic measurements, mice, urinary corticosterone

## Abstract

Mice are used extensively in physiological research. Automated home‐cage systems have been developed to study single‐housed animals. Increased stress by different housing conditions might affect greatly the results when investigating metabolic responses. Urinary corticosteroid concentration is considered as a stress marker. The aim of the study was to compare the effects of different housing conditions and an automated home‐cage system with indirect calorimetry located in an environmental chamber on corticosterone levels in mice. Male mice were housed in different conditions and in automated home‐cage system to evaluate the effects of housing and measuring conditions on urine corticosterone levels. Corticosterone levels in single‐housed mice in the laboratory animal center were consistently lower compared with the group‐housed mice. Single‐housed mice in a separate, small animal unit showed a rise in their corticosterone levels a day after they were separated to their individual cages, which decreased during the following 2 days. The corticosterone levels of group‐housed mice in the same unit were increased during the first 7 days and then decreased. On day 7, the corticosterone concentrations of group‐housed mice were significantly higher compared with that of single‐housed mice, including the metabolic measurement protocol. In conclusion, single housing caused less stress when compared with group‐housed mice. In addition, the urine corticosterone levels were decreased in single‐housed mice before the metabolic measurement started. Thus, stress does not affect the results when utilizing the automated system for measuring metabolic parameters like food and water intake and calorimetry.

## Introduction

Mice are the most often used mammals in biomedical research. It is known that improper housing conditions can be harmful to animals, thus affecting the experimental results by stress, for example (Olsson and Westlund 2007; Arndt et al. 2009; Bartolomucci et al. 2009). To lower exposure to stress in laboratory animals, sufficient space, nesting, and hiding materials should be taken into account during maintenance in the laboratory conditions (Sherwin and Nicol 1997; Van de Weerd et al. 1997; Van Loo et al. 2001).

It has been shown that single housing of male mice does not increase corticosterone level compared to that of group‐housed male mice (Bartolomucci et al. 2003; Hunt and Hambly 2006). This finding can be explained by the lack of the territorial aggression due to social housing conditions and increased space in single housing. As a result of their territoriality and male intolerance against same gender, group housing appears to become a stress factor for animals, which then further affects their behavior and experimental results. However, to the best of our knowledge, the impact of the protocol used in single‐mouse–automated home‐cage recordings in the temperature‐controlled environmental chamber on the urine corticosterone levels as stress marker in mice is not known. The role of the environmental chamber is interesting, since the machine produces constant noise from a compressor, which may also have unwanted effects on the stress levels during measurements. Finally, for the accuracy of animal monitoring single housing is necessary.

Plasma glucocorticoid concentration is rapidly increased when the hypothalamic–pituitary–adrenal axis is stimulated, and therefore considered as a good parameter to assess the stress levels of animals (Bamberg et al. 2001; Touma et al. 2003). Glucocorticoids are secreted into the blood when an animal experiences discomfort or distress (Hunt and Hambly 2006). Cortisol and corticosterone are used as parameters for stress in laboratory animals. In mice, the serum cortisol and corticosterone levels follow circadian rhythm with acrophase during day and low levels during active period in night (Gong et al. 2015). Measurements of plasma glucocorticoid is the most precise and sensitive method to analyze the stress levels in animals, but blood sampling may cause an additional stress leading to rapid rise in glucocorticoids (Harper and Austad 2001; Touma et al. 2003; Hunt and Hambly 2006). The delay of the increase in urinary excretion of corticosterone has been reported within 3 h, indicating that short period of handling should not cause any rise to the urinary corticosterone levels during sampling (Bamberg et al. 2001). Corticosterone, the major glucocorticoid in adult mice (Touma et al. 2003), is excreted into urine and feces and hence in this study noninvasively collected urine samples could be used as stress marker.

To be able to perform good‐quality physiological and behavioral measurements, it is important to maintain the mice as undisturbed as possible and all stress factors should be avoided. Our focus has been to utilize an automated physiological and behavioral monitoring system with drinking and feeding baskets and indirect calorimetry in small laboratory animals and different cage sizes. The study protocol included housing the individual mice in single cages before and during the measurements. In addition, the cages were kept in an environmental chamber, which makes noise to the background and might be considered as a stress factor (Kim et al. 2008). The study protocols were planned to mimic the normal experimental procedures as closely as possible. We investigated the effects of housing style, cage size and monitoring measurements in an environmental chamber by determining the urinary corticosterone levels.

## Materials and Methods

### Animals

Animal experiments were conducted in accordance with the guidelines set by the European Community Council Directives 86/609/EEC and the protocols were approved by Institutional Animal Care and Use Committee of the Provincial Government.

**Figure 1:**
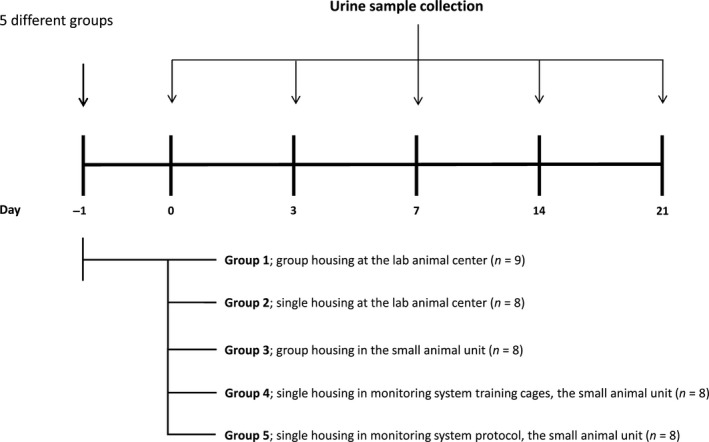
The experimental protocol.

Inbred C57BL/6NCRl male mice were purchased from the laboratory animal center of Oulu University, Finland, at the age of 6–8 weeks. The strain was selected, since it is the most studied strain in our facilities and it is the most commonly used mouse strain in obesity research. All mice used in the present study were originated from the barrier unit of the laboratory animal center (lab animal center) where the animals were bred. The mice pups were weaned from the dams at the age of 5 weeks and divided into separate groups of males and females. At 6–8 weeks of age the mice were transferred from the barrier to the lab animal center located in the same unit. Male mice were divided into five groups (Fig. 1). Group 1 was housed in a group of animals at the lab animal center (*n* = 9). Group 2 mice were housed in individual cages in the same room as the group‐housed mice (*n* = 8). These mice were separated into individual cages upon the transfer to the lab animal center from the barrier. Group 3 was housed in a group condition (*n* = 8) at a separately located small animal unit used for animal monitoring. All the mice studied in the small animal unit were transferred there directly from the barrier with a hooded trolley. If the mice were to be studied in a single‐housed manner, they were separated from groups to single at arrival (Groups 4 and 5). This unit didnot have any additional animals. Prior to the measurements of metabolic parameters, the mice were acclimatized for similar conditions to those during the measurement. Group 4 mice were housed in single in small training cages (*n* = 8) for 3 weeks. Group 5 mice were housed in single in the small training cages for 7 days and in the monitoring system cages for 14 days (*n* = 8). Both types of cages include special water and food containers. The cage sizes of Groups 1 and 2 were 375 mm (L) ×205 mm (W) ×150 mm (H) and 310 mm (L) ×155 mm (W) ×140 mm (H), respectively. Group 3 mice were housed in similar cage than Group 1. The cage size of Groups 4 and 5 was approximately 240 mm (L) ×150 mm (W) ×140 mm (H).

Mice were kept at 21 ± 2°C and relative air humidity was 40–60% and had 12‐h light rhythm with lights on at 6 am with increasing and decreasing dim light periods before lights on and off, respectively. Mice were fed ad libitum commercial rodent chow (Teklad Global Rodent diet T.2018C.12, Harlan Teklad, USA) and had free access to tap water. Aspen bedding and activity blocks were used in the cages during the metabolic measurements (Tapvei^®^, Estonia). For the group‐housed mice, nesting material (PM90L/R, Tapvei^®^, Estonia) was added to the cage. In the lab animal center and in the small separate animal unit only specified trained personnel took care of the mice. The daily animal inspection covered the inspection of fur (smoothness and shininess, possible barbering, or wounds) and behavioral inspection (i.e., normal drink and food intake, appearance of abnormal behavior indicating problems in the well‐being).

### Physiological and behavioral data

For measuring food and water intake and physical activity, an automated indirect open circuit calorimetry placed in environmental cabinet was utilized (LabMaster; Environmental chamber, WB 2000 KHL, TSE Systems GmbH, Germany). The animal monitoring device is located in the small animal unit. Inside the environmental cabinet the temperature was set at 21 ± 1°C and humidity at 60 ± 2.5%. The reported interior noise level is less than 50 dB. The calorimetry components are operated as an open circuit measuring system for the determination of O_2_ consumption, CO_2_ production, respiratory exchange rate (RER), and heat. The activity is detected via infrared sensors. Mice were acclimatized for 1 week for the experimental conditions in training cages that included aspen bedding and activity block. The experimental cages are similar to that of the training cages, except for the lid which is air sealed in the experimental version. During the first week the mice were trained for the special drinking and feeding containers. The drinking bottle allowed access to water only when the mouse was pressing the nipple of the bottle.

### Urine collection for corticosterone and creatinine measurements

Urine collection was performed according to the Fitchett's method (Fitchett et al. 2005). Briefly, one mouse at a time was transferred into a clean empty cage, and samples were collected after the animal urinated. Urine sample (2 × > 10 *μ*L) was collected from the cage by pipette and stored in 1.5 mL micro tubes at −70°C until analysis.

### Establishment of the circadian rhythm of the mice

To confirm the relationship between the circadian rhythm and urinary corticosterone levels of mice in our animal facilities, we first collected the urine samples at 7 am, 1 and 7 pm and 1 am. Sample collection time points were settled according to a previous article demonstrating circadian rhythm of serum cortisol and corticosterone levels in mice (Gong et al. 2015). The mice of this experiment were a separate group (*n* = 3) than used in the later experiments.

### Urine collection of the mice during different housing conditions

The samples were collected at 7 am. In the lab animal center, prior to the start of the sample collection, the mice were acclimatized over 1 week. In the small animal unit, the first urine samples were collected 1 day after the mice were transferred to each housing condition. The first sampling day was set as day 0, and samples were collected at days 0, 3, 7, 14, and 21. In addition to corticosterone, urine creatinine levels were also analyzed to correct the urine corticosterone concentration for differences in glomerulus filtration rate and hydration status, and to assure normal kidney function (Fitchett et al. 2005). The time that was used for sampling per mouse varied from 1 to 60 min. It must be noted that for most of the samples the collection time was closer to 1 than 60 min. The body weights were measured during each sample collection.

### Urine sample analysis

Urine samples were diluted 50‐fold with distilled water and corticosterone levels were measured with ELISA kits according to the manufacturer's instructions (Corticosterone ELISA kit, Enzo Life Sciences Inc., Farmingdale, NY). The intra‐assay variations reported for low, medium, and high corticosterone concentrations were 8.0%, 8.4%, and 6.6%, respectively, and the interassay variations were 13.1%, 8.2%, and 7.8%, respectively. Urine creatinine levels were analyzed by QuantiChrom^™^ Creatinine Assay kit (BioAssay Systems, Hayward, CA) according to the manufacturer's instructions.

### Statistical analysis

Data are represented as means ± standard errors of means (SEM). Multigroup comparisons were conducted by one‐way or two‐way ANOVA followed by Tukey's multiple comparisons test and differences between two group means were compared by Student's *t* test (GraphPad Prism 6, Graph Pad Software Inc, CA). Values of *P *<* *0.05 were considered statistically significant.

## Results

### The effect of circadian rhythm on urine corticosterone levels in mice

**Figure 2:**
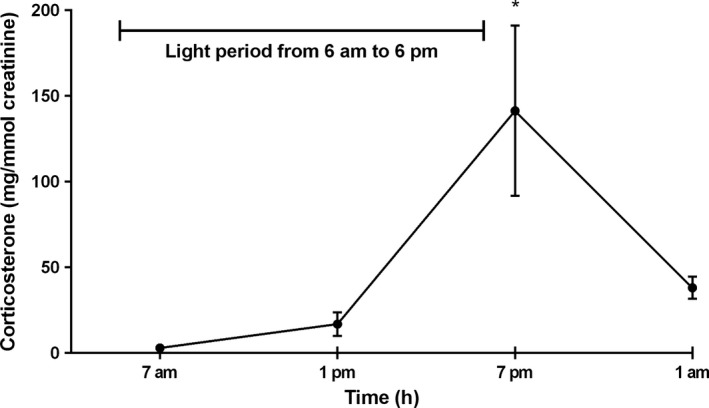
Effect of circadian rhythm on urine corticosterone levels. Corticosterone level (mg/mmol creatinine) of mice urine at different time points in a day. Each value represents the mean ± SEM for three mice. Value with * is significantly different at *P *<* *0.05 by one‐way ANOVA followed with Tukey's multiple comparisons test.

The concentration of urine corticosterone was lowest at 7 am, and it increased toward evening, as expected (Fig. 2). The highest urine corticosterone level was at 7 pm (Fig. 2). The urine corticosterone level at 7 pm was significantly higher than that of at 7 am and 1 pm. Based on these results, which demonstrate normal circadian rhythm, the sampling time for the following experiment was set at 7 am.

### The effect of different housing conditions and metabolic parameters measurement on urinary corticosterone in mice

**Figure 3:**
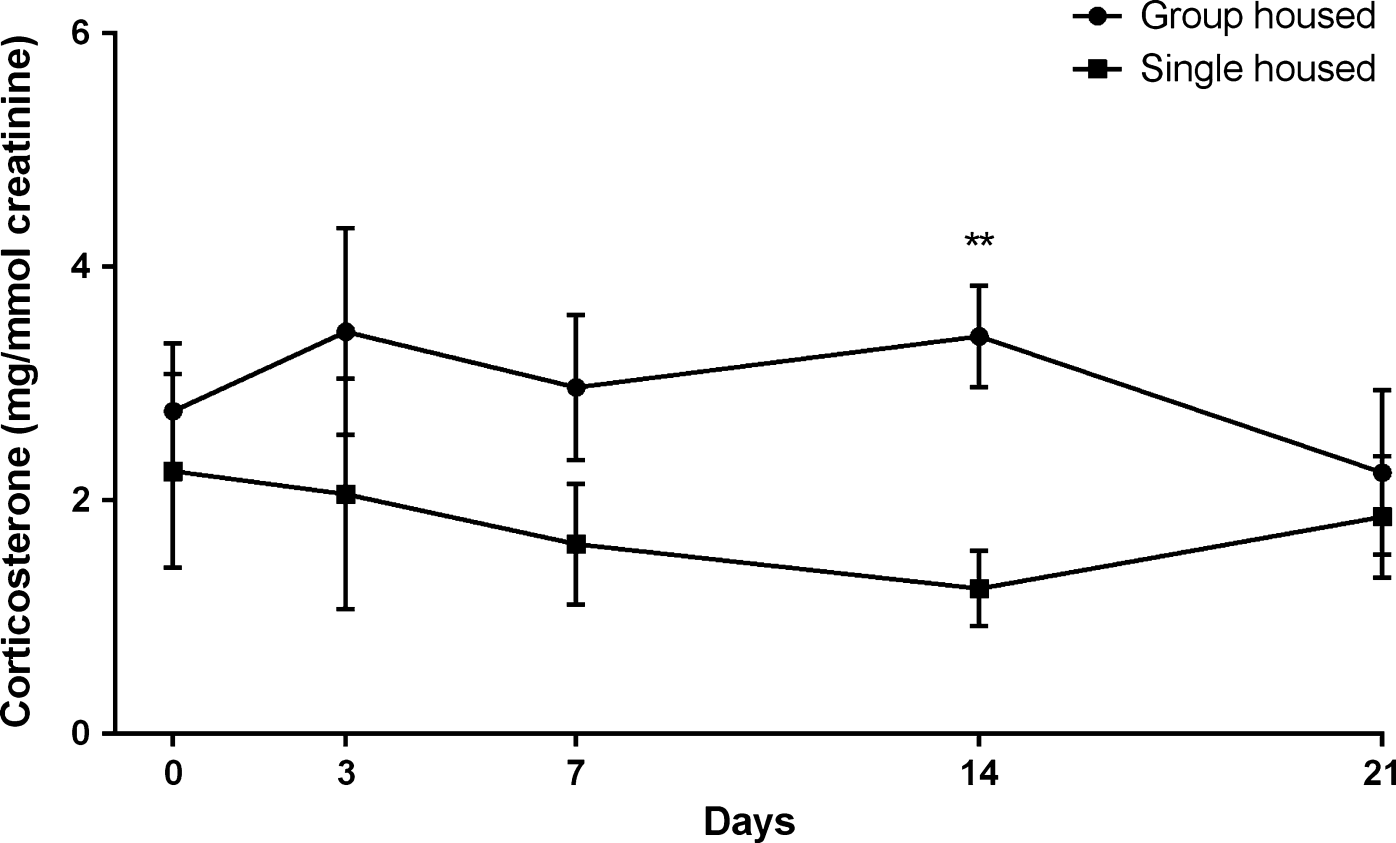
The effect of different housing condition on urine corticosterone levels. Urine corticosterone level (mg/mmol creatinine) of different housing condition of mice. Each value represents the mean ± SEM for nine (group) and eight (single) mice. Value with *** is significantly different at *P *<* *0.01 by Student's *t* test.

The urine corticosterone level was lowest in the morning (Fig. 2) which was consistent with the previous knowledge (Gong et al. 2015), hence it was decided to collect urine samples at 7 am in the experiment. The urine corticosterone levels of Group 1 (group housing at the lab animal center) and Group 2 (single housing at the lab animal center) are shown in Figure 3. The group‐housed mice had consistently higher levels of corticosterone compared with single‐housed mice and at day 14 the corticosterone levels of group‐housed mice were significantly higher than that of single‐housed mice (3.4 ± 0.44 mg/mmol creatinine vs. 1.2 ± 0.32 mg/mmol creatinine).

**Figure 4:**
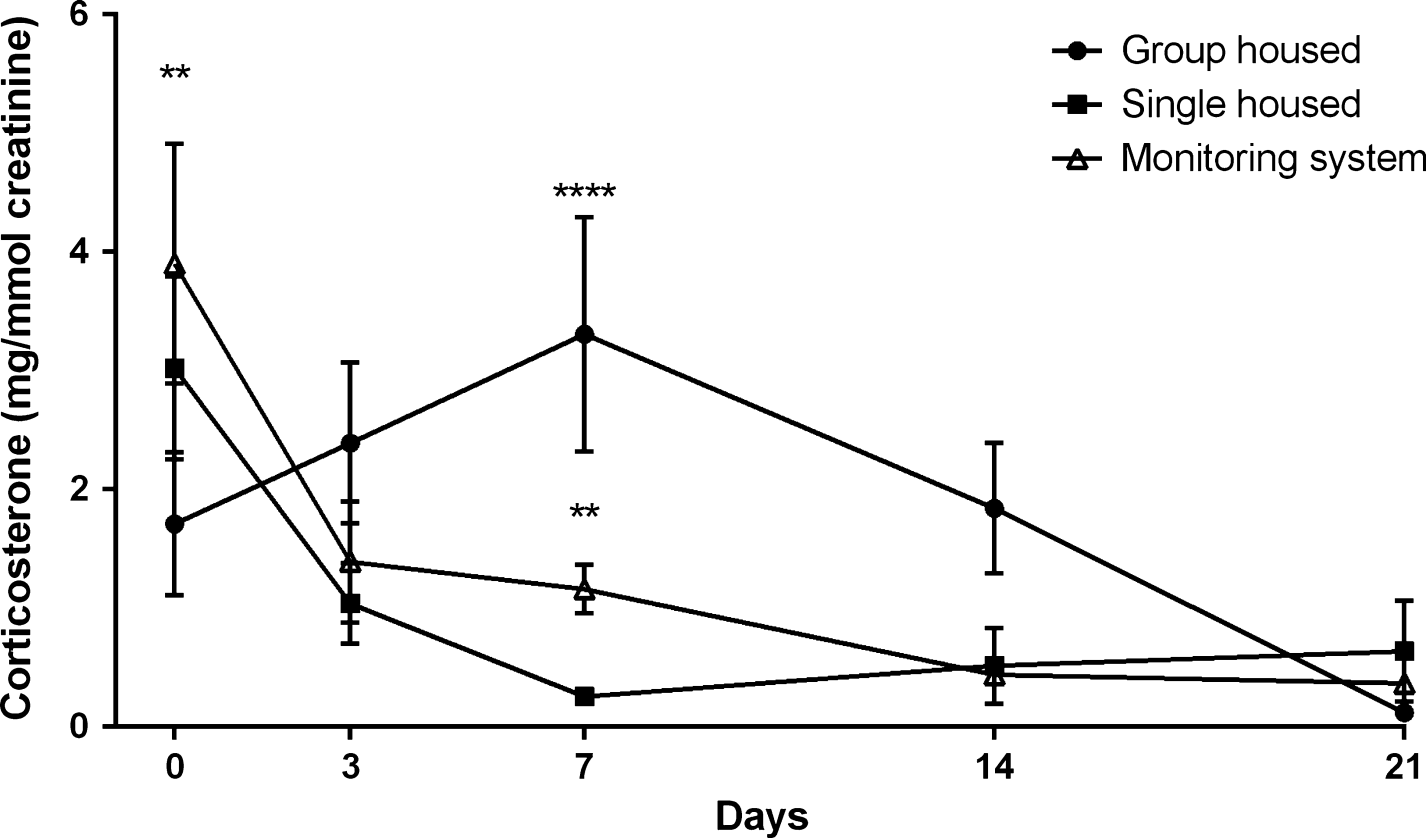
Effect of different housing condition and measuring metabolic performance in an environmental chamber on mice stress. Urine corticosterone level (mg/mmol creatinine) of different housing condition of mice. Each value represents the mean ± SEM for eight mice. At day 0, **P <* *0.01 for group housed versus monitoring system; at day 7, **P <* *0.01 for group housed versus monitoring system and **P <* *0.0001 for group housed versus single housed.

**Figure 5:**
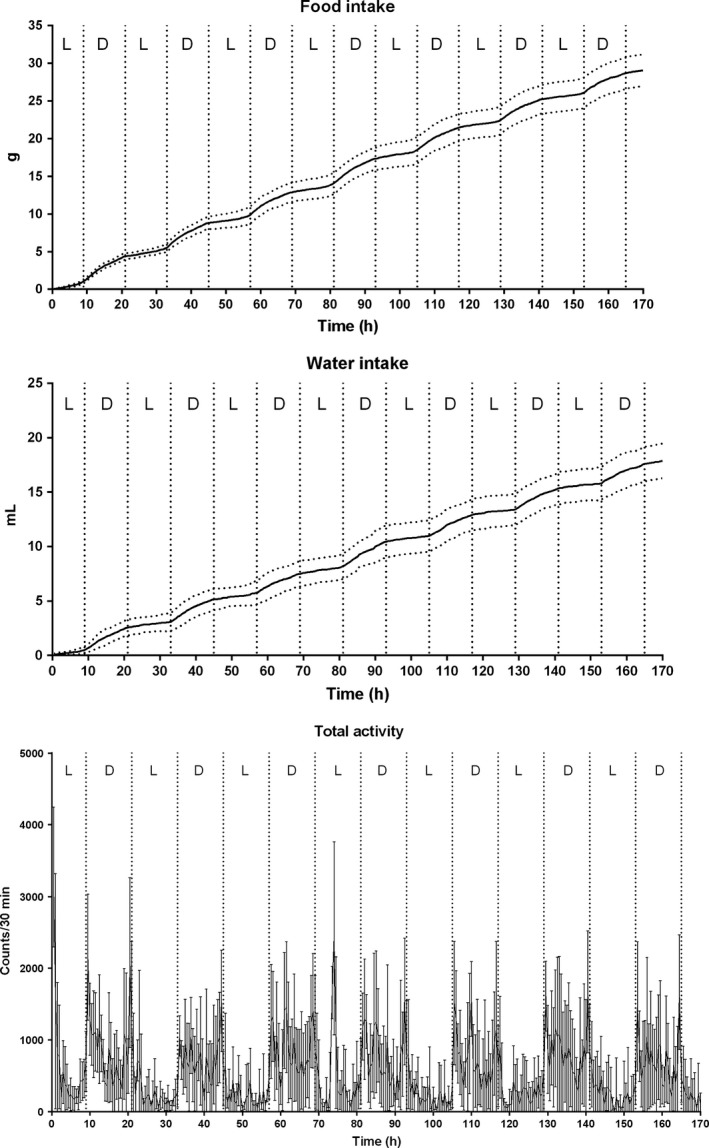
Physiological and behavioral measurements demonstrated normal pattern for food and water consumption and total activity (*n *=* *8, mean ± SEM). In all figures, the light (L) and dark (D) periods are separated with dashed lines.

**Figure 6:**
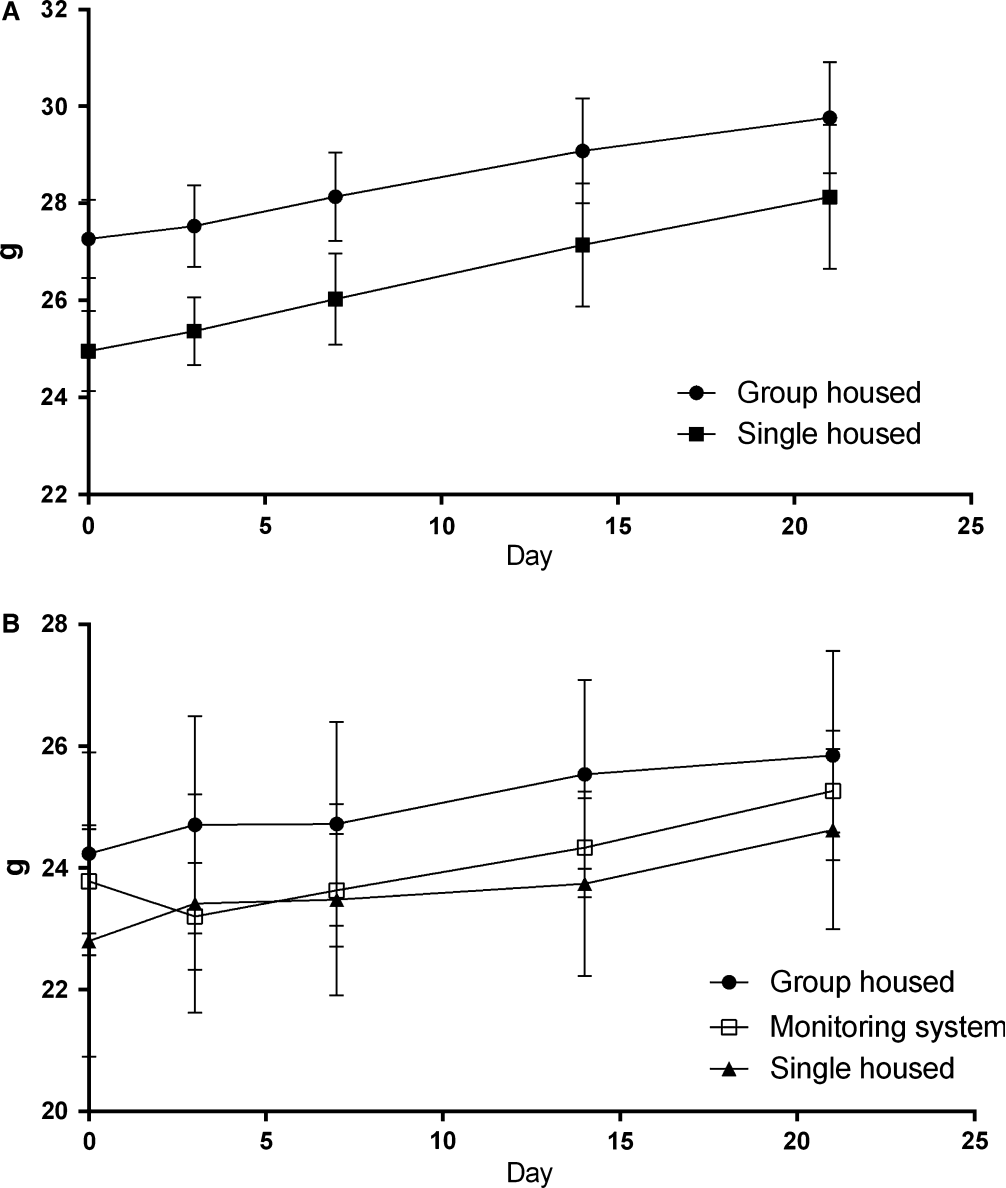
Body weights of mice with different housing conditions. (A) Body weights of group‐housed and single‐housed mice in laboratory animal center. (B) Body weights of group‐housed, monitoring system, and single‐housed mice.

The corticosterone levels of Group 3 (group housing in the small animal unit), Group 4 (single housing in the training cage), and Group 5 (single housing in the training cage for 7 days and in the monitoring system for 14 days with experimental protocol) are shown in Figure 4. The corticosterone levels of group‐housed mice were increased during the first 7 days and decreased gradually during the experiment. On the other hand, the corticosterone levels of single‐housed mice in the monitoring system and small cages were increased at the beginning of the study and then decreased during the first 3 days and remained lower during the rest of the experiment. The animal monitoring revealed normal behavior for drink and food intake as well as locomotor activity (Fig. 5). On the day 7 (Fig. 4), the corticosterone concentrations of group‐housed mice were significantly higher compared with single‐housed mice (3.3 ± 0.99 mg/mmol creatinine [Group 3], 0.3 ± 0.05 mg/mmol creatinine [Group 4], and 1.2 ± 0.21 mg/mmol creatinine [Group 5]) in the small cages and monitoring system. Initial body weights in the beginning of the study were significantly different (*P *<* *0.0001; one‐way ANOVA) between the groups. There were no significant differences in the body weight gain between the investigated groups (Fig. 6a,b).

## Discussion and Conclusions

The present study demonstrated that the urine corticosterone concentration was lowest in the morning and increased toward the evening when the mice become physically active confirming the normal circadian rhythm in the mice. Thereafter the corticosterone levels decreased toward the morning. In single‐housed mice at the small animal unit, the first measured corticosterone concentrations were higher than those in the group‐housed mice suggesting that the separation of the mice from a group to single housing 1 day before the first corticosterone measurement, or the introduction of novel feeding and drinking equipment caused a stress response. Physical activity, food intake, and water consumption of the group in animal monitoring was normal. The present results demonstrated that there is no adverse effects of single housing in smaller cage size. Also group housing and automated monitoring measurements in the environmental chamber did not cause stress when the urinary corticosterone is used as a stress marker. The automated monitoring system significantly differs from metabolic monitoring system with grid floor which has been earlier shown to cause stress to mice (Kalliokoski et al. 2013). In addition, single housing may have behavioral effects in some mouse strains like DBA/2 (Võikar et al. 2005).

Corticosterone is released from the adrenal cortex when the hypothalamic–pituitary–adrenal axis is activated in response to stimulus such as stress (Ronald de Kloet 2000). It is an established stress marker of animals especially in rodents (Brown and Grunberg 1995; Touma et al. 2003; Arndt et al. 2009). Measuring corticosterone in plasma sample is the most common way to analyze the stress level of laboratory animals. However, plasma corticosterone level responds sensitively to stressors and blood sampling is itself invasive and a stressful technique particularly for small animals (Touma et al. 2003; Hunt and Hambly 2006). We chose to collect urine sample for corticosterone assay because urine corticosterone secretion does not respond to acute stress and the excretion of corticosterone, produced during sampling to urine takes several hours (Bamberg et al. 2001).

In general, mice and rats are recommended to be housed in groups. According to the DIRECTIVE 2010/63/EU, which is for the protection of animals for scientific purposes, laboratory animals, except those which are naturally solitary, should be housed in groups and the duration of single housing due to the experiment should be kept as short as possible. These guidelines are based on the knowledge that social isolation and lack of social support have negative effects on many mammalian species (Valzelli 1973; Brown and Grunberg 1995; Lyons et al. 1999; te Ruis et al. 2001). In general, group housing is recommended to social animals, such as mice and rats, to maximize their well‐being (Olsson and Westlund 2007; DIRECTIVE 2010/63/EU). Nevertheless, in certain study settings single housing is necessary to obtain individual results. For example, to study the effect of lack of social stimuli or to monitor individual parameters, or to protect animals from aggression by dominant partner, single housing is required at least in laboratory conditions. These guidelines which advice group housing, are based on the knowledge that social isolation and absence of social support have disadvantageous effects on many mammalian species (Valzelli 1973; Brown and Grunberg 1995; Lyons et al. 1999; te Ruis et al. 2001).

Consistent with our present results, several studies have shown that single housing does not increase stress markers in mice compared with group housing (Hunt and Hambly 2006; Arndt et al. 2009). The present study demonstrated that single housing in normal and small size cages with or without animal monitoring in an environmental chamber do not have adverse effect on stress level of mice in comparison to group housing. On the contrary, we observed that group‐housed mice showed significantly higher corticosterone levels during the experimental period. The reason for the high corticosterone may be aggression and/or cage size. In group‐housed male mice, the mice usually perform aggressive behavior to develop and maintain social hierarchy (Van Loo et al. 2003). The age of the mice in the present study was set to 6–8 weeks, since they are considered to be full grown, mature, and commonly investigated at this age in the calorimetric studies. Another possible explanation of the present result is cage size and the area per mouse. Van Loo et al. (2001) showed that the aggression of male mice during group housing can be affected by the cage size. However, when the cage size is as low as 80 cm^2^/mouse, it could cause stress due to crowding. In the current study, the density of Group 1 (group housing at the lab animal center) mice was 85.4 cm^2^/mouse and Group 3 (group housing in small animal unit) mice was 96 cm^2^/mouse, thus the higher density might become a stress factor.

In conclusion, we adopted urinary corticosterone as a stress marker and demonstrated that the protocol, which is used for animal monitoring in an environmental chamber, does not induce lasting stress in male C57Bl/6 mice. In addition, the smaller cage size, which is required by the calorimetry equipment, or single housing do not induce stress reaction.

## Conflict of Interest

None declared.
